# Mast Cell-Specific Expression of Human Siglec-8 in Conditional Knock-in Mice

**DOI:** 10.3390/ijms20010019

**Published:** 2018-12-21

**Authors:** Yadong Wei, Krishan D. Chhiba, Fengrui Zhang, Xujun Ye, Lihui Wang, Li Zhang, Piper A. Robida, Liliana Moreno-Vinasco, Ronald L. Schnaar, Axel Roers, Karin Hartmann, Chang-Min Lee, Delia Demers, Tao Zheng, Bruce S. Bochner, Zhou Zhu

**Affiliations:** 1 Section of Allergy and Clinical Immunology, Yale University School of Medicine, New Haven, CT 06511, USA; ywei16@jhu.edu (Y.W.); fengrui.zhang@yale.edu (F.Z.); wdxjy@whu.edu.cn (X.Y.); wanglh10318@163.com (L.W.); syszhang@hotmail.com (L.Z.); tao_zheng@brown.edu (T.Z.); 2 Department of Medicine, Division of Allergy and Immunology, Northwestern University Feinberg School of Medicine, Chicago, IL 60611, USA; krishan.chhiba@northwestern.edu (K.D.C.); piper.wedman@northwestern.edu (P.A.R.); lmoreno@deptofmed.arizona.edu (L.M.-V.); bruce.bochner@northwestern.edu (B.S.B.); 3 Department of Pharmacology, Johns Hopkins University School of Medicine, Baltimore, MD 21224, USA; schnaar@jhu.edu; 4 Institute of Immunology, University of Technology Dresden, 01069 Dresden, Germany; Axel.Roers@tu-dresden.de; 5 Department of Dermatology, University of Lübeck, 23538 Lübeck, Germany; Karin.Hartmann@uksh.de; 6 Department of Molecular Microbiology and Immunology, Department of Pediatrics, Brown University Alpert Medical School, Providence, RI 02912, USA; chang-min_lee@brown.edu (C.-M.L.); delia_demers@brown.edu (D.D.)

**Keywords:** allergic disease, mouse mast cells, ROSA26, Siglec-8

## Abstract

Sialic acid-binding Ig-like lectin 8 (Siglec-8) is expressed on the surface of human eosinophils, mast cells, and basophils—cells that participate in allergic and other diseases. Ligation of Siglec-8 by specific glycan ligands or antibodies triggers eosinophil death and inhibits mast cell degranulation; consequences that could be leveraged as treatment. However, Siglec-8 is not expressed in murine and most other species, thus limiting preclinical studies in vivo. Based on a ROSA26 knock-in vector, a construct was generated that contains the CAG promoter, a LoxP-floxed-Neo-STOP fragment, and full-length Siglec-8 cDNA. Through homologous recombination, this Siglec-8 construct was targeted into the mouse genome of C57BL/6 embryonic stem (ES) cells, and chimeric mice carrying the ROSA26-Siglec-8 gene were generated. After cross-breeding to mast cell-selective Cre-recombinase transgenic lines (CPA3-Cre, and Mcpt5-Cre), the expression of Siglec-8 in different cell types was determined by RT-PCR and flow cytometry. Peritoneal mast cells (dual FcεRI^+^ and c-Kit^+^) showed the strongest levels of surface Siglec-8 expression by multicolor flow cytometry compared to expression levels on tissue-derived mast cells. Siglec-8 was seen on a small percentage of peritoneal basophils, but not other leukocytes from CPA3-Siglec-8 mice. Siglec-8 mRNA and surface protein were also detected on bone marrow-derived mast cells. Transgenic expression of Siglec-8 in mice did not affect endogenous numbers of mast cells when quantified from multiple tissues. Thus, we generated two novel mouse strains, in which human Siglec-8 is selectively expressed on mast cells. These mice may enable the study of Siglec-8 biology in mast cells and its therapeutic targeting in vivo.

## 1. Introduction

Sialic acid immunoglobulin-like lectin 8 (Siglec-8) is a member of the CD33 subfamily of glycan receptors called Siglecs. Siglec-8 is unique in its expression on human eosinophils, mast cells, and basophils, which are important innate immune effector cells in allergy, asthma, and other type 2 disorders [1,2,3,4]. Polymorphisms in the Siglec-8 gene are associated with asthma susceptibility [5]. It has been demonstrated that the engagement of Siglec-8 with specific antibodies or sialylated, sulfated glycan ligands led to death in eosinophils, while inhibition of the FcεRI-mediated mediator release by mast cells was observed without any effect on their survival [6,7,8,9,10]. In mice, Siglec-F on eosinophils is considered a functional paralog of Siglec-8 because of some similarities in expression patterns, recognition of glycan ligands, and regulatory functions [11,12]. However, there are some noticeable differences between Siglec-8 and Siglec-F, including patterns of expression on other cells (e.g., Siglec-F is not expressed by mouse mast cells), additional sialylated, non-sulfated ligands recognized by Siglec-F, and more modest death responses seen upon Siglec-F engagement [3,13].

Mast cells play important roles in allergic diseases and asthma, and mast cell abnormalities in number and function underlie disorders such as mastocytosis, urticaria and the mast cell activation syndrome [14,15,16]. Siglec-8 expression was found on human mast cells from several organs and some mast cell lines [10,17,18]. To date, all studies on Siglec-8 have been carried out using human tissues, cells, and cell lines in vitro, in large part because Siglec-8 is not expressed in lower species other than some non-human primates [18]. Recently, our group generated a new mouse strain in which Siglec-8 expression was specifically targeted into eosinophils [19]. This will allow further studies of Siglec-8 in eosinophils in vivo, particularly in the context of disease models and therapeutic potentials. However, animal models in which Siglec-8 is specifically expressed on mast cells are still not available. In this work, using the Cre-LoxP system, we generated two new mouse strains that were engineered to express Siglec-8 selectively on mouse mast cells, and we performed the initial characterization of Siglec-8 surface expression and mRNA levels, consistency of mast cell selectivity of expression, and the impact of its expression on mast cell numbers in tissues.

## 2. Results

### 2.1. In Vitro Testing of ROSA26-Siglec-8 Constructs in HEK 293T Cells

**Figure 1:**
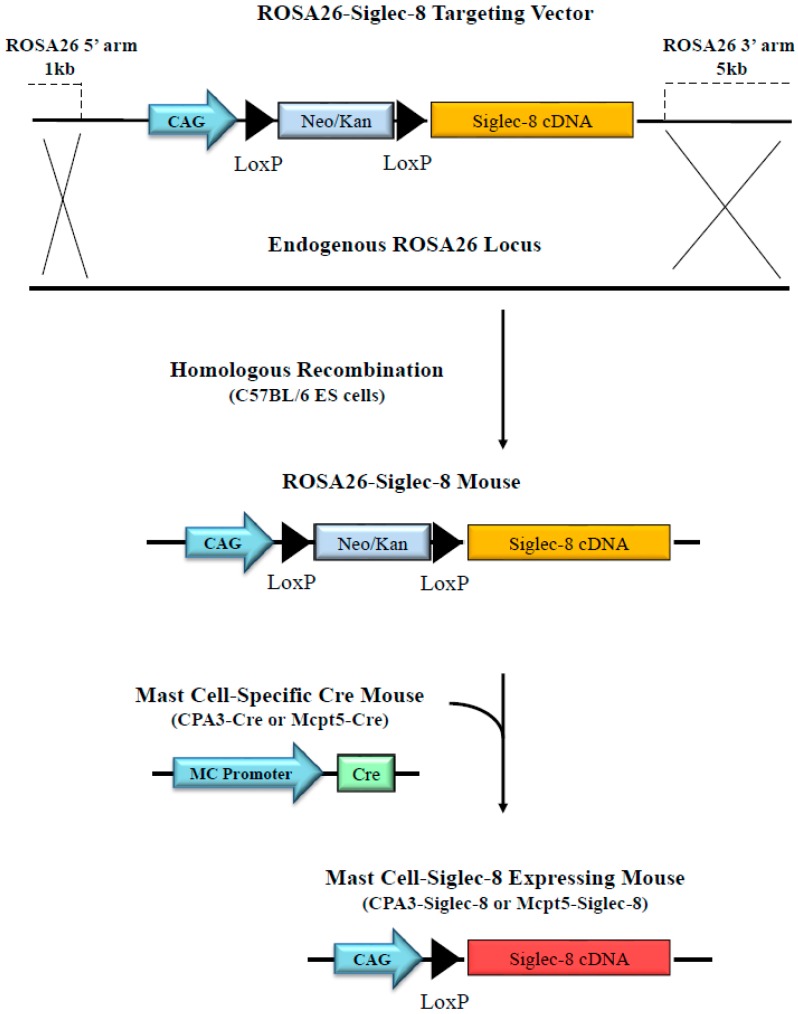
Targeted expression of human Siglec-8 into mouse mast cells. Schematic overview of the generation of conditional knock-in mice that express human Siglec-8 on mast cells. A ROSA26-DEST vector-based targeting construct was generated that contains the CAG promoter, LoxP-floxed-Neo/Kan as a STOP fragment followed by human Siglec-8 cDNA. Using standard procedures, ROSA26-CAG-STOP-Siglec-8 (or simply ROSA26-Siglec-8) knock-in mice were generated. After cross-breeding to mast cell specific Cre mouse lines (CPA3-Cre and Mcpt5-Cre), Cre-mediated removal of the conditional Neo-STOP fragment led to CAG promoter driven human Siglec-8 expression on mast cells. Terms used are CAG: CAG promoter (a combined fusion of CMV enhancer, chicken beta-Actin promoter and rabbit beta-Globin splice acceptor site); Neo/Kan: Neomycin/Kanamycin resistant gene; LoxP: LoxP recognition sequences.

**Figure 2:**
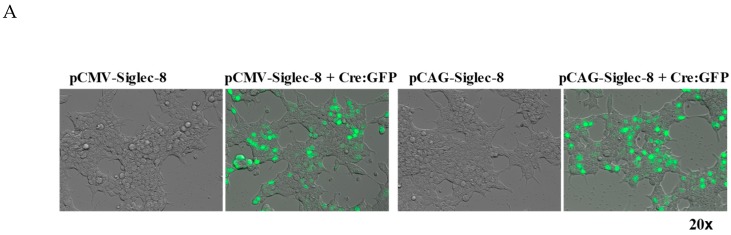

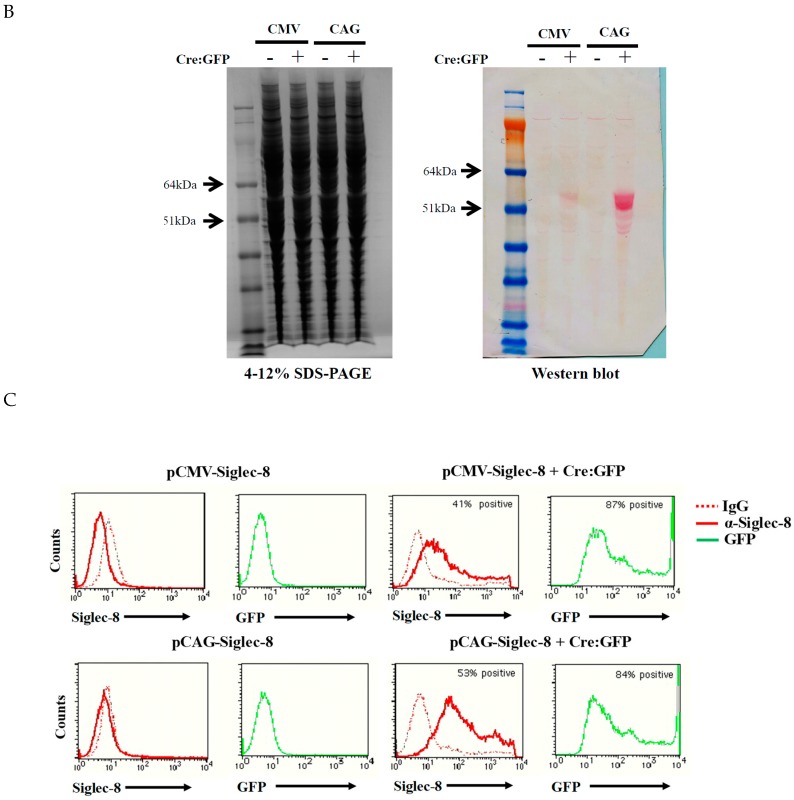
In vitro testing of conditional expression of human Siglec-8 in transfected HEK 293T cells. (**A**) ROSA26-CMV-Siglec-8 and ROSA26-CAG-Siglec-8 conditional knock-in targeting constructs were transfected with or without the Cre:GFP plasmid into confluent HEK 293T cells. Twenty-four hours after transfection, cells were examined and photographed under fluorescent microscope. The presence of GFP indicated successful transfection and expression of the Cre:GFP plasmid; (**B**) total cellular proteins from each sample were harvested and subjected to 4–12% gradient SDS-PAGE and analyzed by western blot using anti-Siglec-8 antibody (Sheep, primary). Anti-sheep IgG conjugated with AP (secondary) was used to visualize the positive bands; (**C**) transfected HEK 293T cells were analyzed by flow cytometry using anti-Siglec-8 antibody. Only cells transfected with ROSA26-CAG-Siglec-8 or ROSA26-CMV-Siglec-8 construct plus the Cre:GFP plasmid showed Siglec-8 expression.

We generated two ROSA26-Siglec-8 targeting constructs, pCAG-Neo-Siglec-8 (Figure 1) and pCMV-Neo-Siglec-8. To verify that the Neo-STOP fragment in these constructs can be removed by Cre, and that the Siglec-8 cDNA gets appropriately expressed on the cell surface, we separately transfected the two constructs into confluent HEK 293T cells with or without co-transfection of the pCAG-Cre:GFP plasmid using Lipofectamine 2000. To confirm efficient transfection, cells were examined 24 h later under a fluorescence microscope. GFP-positive cells indicated efficient transfection and expression of GFP and Cre (Figure 2A). Western blot analysis was done using the anti-Siglec-8 antibody, and showed that cells transfected with pCMV-Neo-Siglec-8 or pCAG-Neo-Siglec-8 without pCre:GFP did not show any Siglec-8 positive bands. In contrast, western blot analysis of cells transfected with either targeting construct plus the pCre:GFP revealed a band at 54 kDa, consistent with the size of the previously described full-length Siglec-8 [20]. Notably, pCAG-Neo-Siglec-8-transfected cells showed a more intense band than cells transfected with pCMV-Neo-Siglec-8 (Figure 2B). We also analyzed Siglec-8 expression on the cell surface using flow cytometry. Only cells transfected with the pCAG-Neo-Siglec-8 or pCMV-Neo-Siglec-8 constructs plus the pCre:GFP construct, showed strong levels of Siglec-8 expression (Figure 2C). These results demonstrated that: (1) The ROSA26 vector-based conditional Siglec-8 targeting constructs were responsive to Cre; (2) Siglec-8 can be conditionally expressed on the cell surface; and (3) between the two constructs, the pCAG-Neo-Siglec-8 construct was more efficient. Thus, this construct was used for the subsequent generation of the ROSA26-Siglec-8 knock-in mice.

### 2.2. Generation of Conditional Knock-in ROSA26-Siglec-8 Mice and Targeting of Siglec-8 Expression to Mast Cells

Using the targeting construct and standard procedures of embryonic stem (ES) cell transfection and blastocyst injection, chimeric mice were generated and genotyped by PCR with Siglec-8 specific primers (data not shown). After breeding to wild type C57BL/6 mice, germline transmission was confirmed and ROSA26-Siglec-8 mice on the C57BL/6 genetic background were obtained. These mice were born at normal Mendelian frequencies and were viable without any obvious phenotype. In order to generate mast cell-targeted Siglec-8-expressing mice, ROSA26-Siglec-8 mice were cross-bred to two different Cre mouse lines, CPA3-Cre [21] and Mcpt5-Cre [22]. From this breeding arrangement, CPA3-Cre-Siglec-8 (CPA3-Siglec-8), and Mcpt5-Cre-Siglec-8 (Mcpt5-Siglec-8) mice were obtained as verified by genotyping. Cre-positive and ROSA26-Siglec-8-positive mice were also identified and used as controls in subsequent experiments, with the genotype of each group indicated in each experiment. Both single- and double-allele Siglec-8 floxed mice were tested and indicated in each experiment.

### 2.3. Equal Numbers of Mast Cells in Tissues of Mcpt5-Siglec-8 and Control Mice

**Figure 3:**
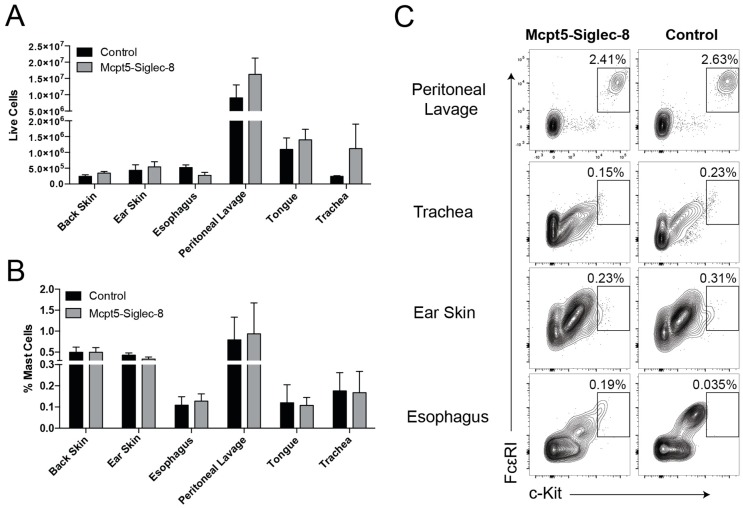
Equal mast cell numbers in control and Mcpt5-Siglec-8 mice across multiple tissues. (**A**) Quantification of total live cells isolated from various tissues; (**B**) average frequency of mast cells (CD45^+^ CD11b^−^ FcεRI^+^ c-Kit^+^ of live cells) in the tissues of Mcpt5-Siglec-8 (Mcpt5-Cre^+/−^ SIG8^+/−^, *n* = 3) and control (*n* = 4: WT, *n* = 1 and Mcpt5-Cre^−/−^ SIG8^+/−^, *n* = 3) mice; and (**C**) representative flow cytometry plots of dispersed tissues showing live CD45^+^ CD11b^−^ cells with a gate for FcεRI^+^ c-Kit^+^ cells. Data in (**A**) and (**B**) are from three independent experiments, and the mean ± SEM of *n* = 3–4 are displayed. No significant differences between the groups were identified (two-way ANOVA).

To determine whether targeting mast cells using the Cre-LoxP system would lead to alterations in mast cell numbers and in their tissue distribution in vivo, we collected cells from multiple tissues and measured the presence and percentage of FcεRI^+^ c-Kit^+^ mast cells using flow cytometry. In Mcpt5-Siglec-8 and control mice equal numbers of CD45^+^CD11b^−^FcεRI^+^c-Kit^+^ mast cells were present in all locations and tissues examined, including peritoneal cavity, trachea, ear skin, and esophagus (Figure 3). These findings indicated that the introduction of Siglec-8 using the Cre-LoxP technology into mast cells did not have any adverse effects on the development and tissue distribution of mast cells in vivo.

### 2.4. Expression of Siglec-8 on Mast Cells and Basophils in CPA3-Siglec-8 Mice on Mast Cells in Mcpt5-Siglec-8 Mice

**Figure 4:**
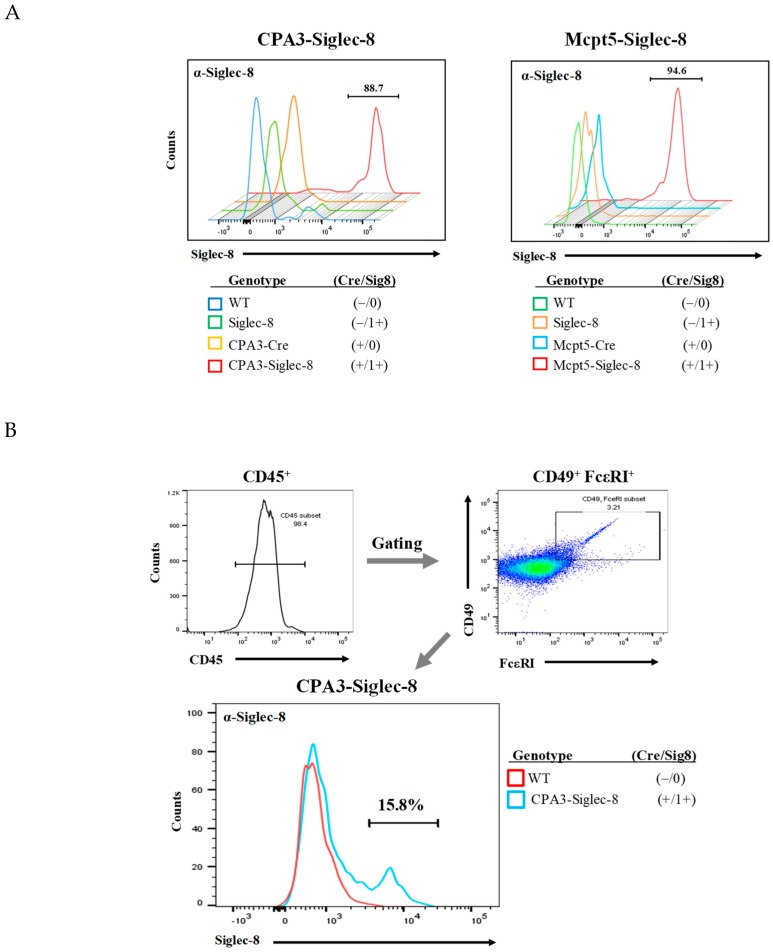
Expression of human Siglec-8 on mast cells and basophils. (**A**) Peritoneal cells were collected from WT (−/0), ROSA26-Siglec-8 (−/1+), CPA3-Cre (+/0) or Mcpt5-Cre (+/0), and CPA3-Siglec-8 (+/1+) or Mcpt5-Siglec-8 (+/1+) mice, and expression of Siglec-8 was determined by flow cytometry using anti-Siglec-8 mAb after gating for CD45^+^FcεRI^+^c-Kit^+^ (CD117) mast cells. Panels are plots of anti-Siglec-8 stained cells from CPA3-Siglec-8 and Mcpt5-Siglec-8 mice and their corresponding controls. The numbers are percentages of anti-Siglec-8 mAb stained cells. Shown are representative results from three independent sets of experiments; (**B**) peritoneal cells from CPA3-Siglec-8 and WT mice were analyzed for Siglec-8 expression on CD45^+^FcεRI^+^CD49b^+^ basophils. Shown are representative results from two separate experiments.

**Figure 5:**
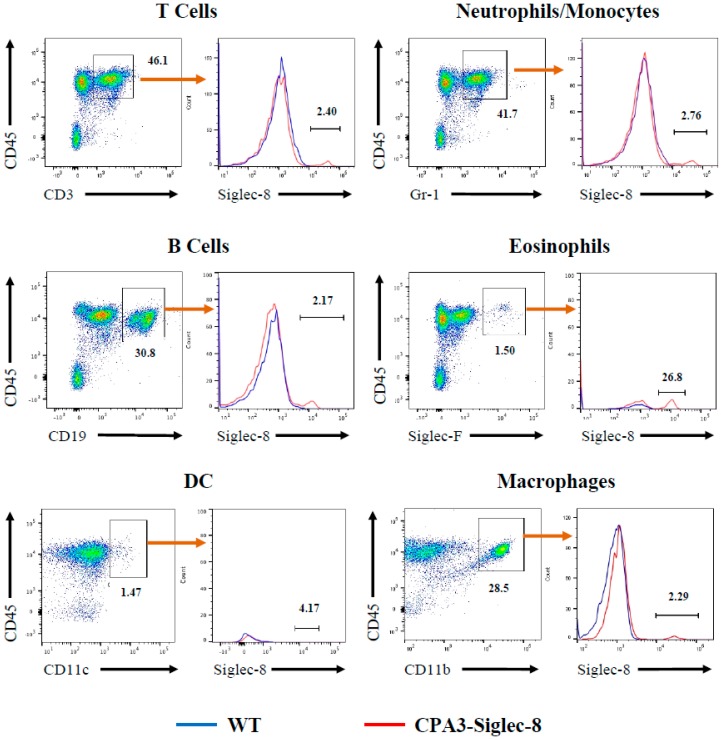
Minimum or no surface expression of Siglec-8 on leukocytes other than mast cells and basophils. Splenocytes were collected from WT and CPA3-Siglec-8 mice and analyzed for Siglec-8 expression after gating to CD45^+^ and specific cell markers, CD3 for T cells, CD19 for B cells, CD11c for dendritic cells (DC), Gr-1 for neutrophils and monocytes, Siglec-F for eosinophils, and CD11b for macrophages. The numbers are percentages of indicated cell populations. Shown are representative plots of three independent experiments.

To determine whether Siglec-8 was correctly targeted to mouse mast cells in vivo, we collected peritoneal cells from CPA3-Siglec-8 mice, Mcpt5-Siglec-8 mice, and their corresponding control groups and measured the expression of Siglec-8 on cells by flow cytometry. As shown in Figure 4A, about 90% of CD45^+^FcεRI^+^c-Kit^+^ mast cells from CPA3-Siglec-8 and Mcpt5-Siglec-8 mice expressed cell surface Siglec-8, whereas all control groups, including WT, Siglec-8 (ROSA26-Siglec-8 KI), CPA3-Cre, and Mcpt5-Cre mice did not. In addition, Siglec-8 expression was found on about 15% of peritoneal basophils from CPA3-Siglec-8 mice, but not on WT basophils (Figure 4B). This is consistent with CPA3 promoter-driven Cre activity and GFP expression in basophils (14%) in the CPA3-Cre transgenic mice as described previously [21]. Furthermore, Siglec-8 expression was not generally detected on other leukocytes. Siglec-8 staining was either barely above background or on a very small subset of cells when splenocytes were analyzed using flow cytometry (Figure 5). These data demonstrate that using two mast cell-specific Cre mouse lines, we have selectively targeted Siglec-8 into mouse mast cells in vivo.

### 2.5. Expression of Siglec-8 on Mast cells and Its Tissue Distribution

**Figure 6:**
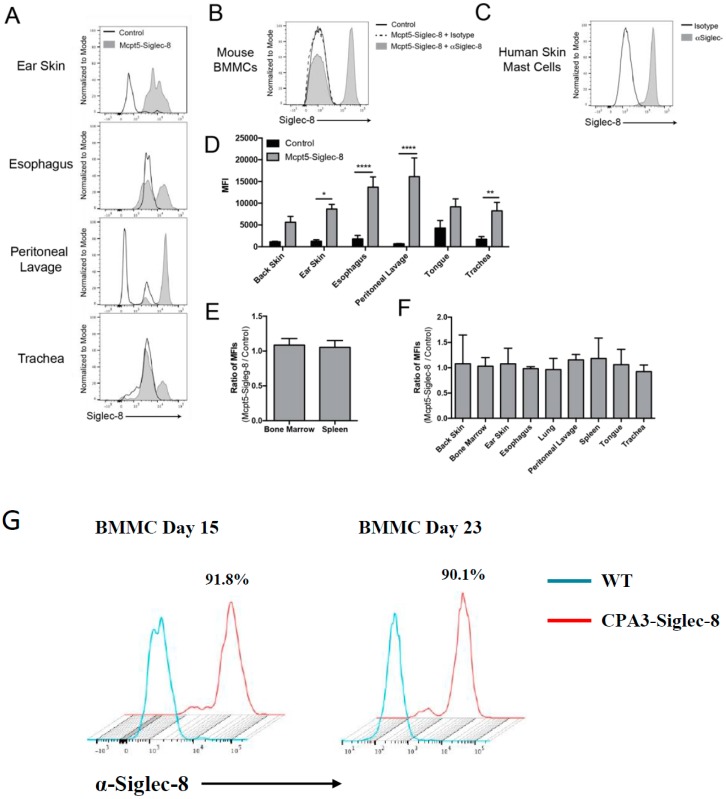
Mast cells selectively express surface Siglec-8 protein in the Mcpt5-Siglec-8 mouse. (**A**) Representative histograms of Siglec-8 expression on mast cells (CD45^+^ CD11b^−^ FcεRI^+^ c-Kit^+^ of live cells) in tissues from Mcpt5-Siglec-8 and control mice. Cells from both mice were stained with anti-Siglec-8 mAb (clone 2E2-AF488) and surface expression of Siglec-8 was assessed by flow cytometry; (**B**) BMMCs from Mcpt5-Siglec-8 and control mice were stained with anti-Siglec-8 mAb or isotype control IgG; (**C**) Human skin-derived mast cells were assessed for surface Siglec-8 expression; (**D**) Quantification of MFI of Siglec-8 staining on mast cells (CD45^+^ CD11b^−^ FcεRI^+^ c-Kit^+^ of live cells) isolated from Mcpt5-Siglec-8 (*n* = 3) and control (*n* = 4: either WT, *n* = 1 or *Mcpt5-Cre^−/−^ SIG8^+/−^, n* = 3) mice. Ratio of Siglec-8 MFI (Mcpt5-Siglec-8/Control) in non-mast cell populations gated for (**E**) eosinophils (live CD45^+^ Siglec-F^mid^), and (**F**) non-immune cells (live CD45^−^); and (**G**) surface expression of Siglec-8 on BMMCs from CPA3-Siglec-8 mice. For panels (**A**–**F**), shown are data from 3 independent experiments, and the mean±SEM of *n* = 3–4 are displayed. Shown in panel G are representative flow cytometry plots of two independent experiments. * *p* < 0.05, ** *p* < 0.01, **** *p* < 0.0001 (two-way ANOVA).

To further determine the expression of Siglec-8 on mast cells in different tissues, we analyzed cells isolated from various tissues of Mcpt5-Siglec-8 and littermate control mice using flow cytometry. As shown in Figure 6A, Siglec-8-expressing CD45^+^CD11b^−^FcεRI^+^c-Kit^+^ mast cells were only found in tissues from Mcpt5-Siglec-8 mice. Interestingly, the ratio of Siglec-8^+^ cells to Siglec-8^−^ cells appeared to be different in the tissues examined. For example, cells from ear skin had the highest ratio of Siglec-8^+^ cells, with peritoneal lavage cells next, followed by cells from the esophagus and trachea (Figure 6A). Bone marrow derived mast cells (BMMCs) were also positive for Siglec-8 (Figure 6B). As a reference, human skin-derived mast cells were assessed for surface Siglec-8 expression with the same anti-Siglec-8 mAb or isotype control, and similar levels of Siglec-8 on mature human skin mast cells were observed (Figure 6C). Looking from another perspective, the mean fluorescent intensity (MFI) of cells from different tissues positive for Siglec-8 staining was quantitated and revealed that cells from control mice had no Siglec-8 staining above background, whereas cells from Mcpt5-Siglec-8 mice had high levels of staining. Particularly, peritoneal lavage cells had the highest MFI, followed by those from esophagus, ear skin, and the rest of tissues examined (Figure 6D). Further analyses of Siglec-8 expression on other cell types in mice showed that the ratios of MFI (Mcpt5-Siglec-8 cells vs. control cells) for non-mast cell populations, including eosinophils (live CD45^+^ Siglec-F^mid^), and non-immune cells (live CD45^−^) were about even, and essentially at the background levels, consistent with the absence of any surface Siglec-8 expression on these various non-mast cell populations (Figure 6E,F). Finally, BMMCs from the CPA3-Siglec-8 mice also uniformly expressed strong levels of Siglec-8 (Figure 6G). These results demonstrated that Siglec-8 was selectively expressed on mast cells in Mcpt5-Siglec-8 mice and in the examined tissues from CPA3-Siglec-8 mice.

**Figure 7:**
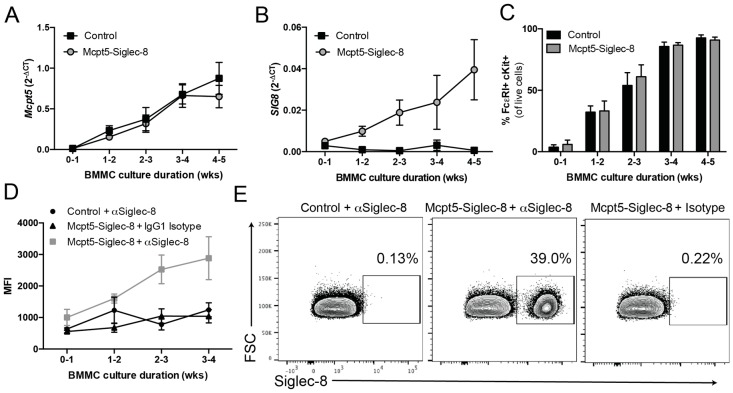
A subset of BMMCs from Mcpt5-Siglec-8 mice express high levels of surface Siglec-8. BMMCs were generated from Mcpt5-Siglec-8 (*n* = 4) and control (*Mcpt5-Cre^−/−^ SIG8^+/−^, n* = 5) mice. (**A**) *Mcpt5* and (**B**) *Siglec-8* mRNA levels were quantified by qPCR weekly during the maturation of the mast cells; (**C**) purity of BMMCs was assessed weekly by flow cytometry showing % FcεRI^+^ c-Kit^+^ of live cells; (**D**) surface Siglec-8 protein expression was determined by flow cytometry; (**E**) representative flow cytometry plots of BMMCs (at four weeks) showing FcεRI^+^ c-Kit^+^ cells and gating for Siglec-8^+^ cells. Data are from three independent experiments, and the mean ± SEM of *n* = 4–5 are displayed.

To further assess Siglec-8 mRNA and surface protein expression on mast cells derived from bone marrow, BMMCs from different groups of mice were cultured. At specified time points, BMMCs from Mcpt5-Siglec-8 mice and control mice were analyzed for Mcpt5 and Siglec-8 mRNA expression by RT-PCR and flow cytometry. First, the expression of the *Mcpt5* gene was determined at the mRNA level, since the expression of Cre and subsequent Siglec-8 expression is dependent on the activity of the Mcpt5 promoter in mast cells in this Cre-LoxP system. As shown in Figure 7A, Mcpt5 mRNA was readily detected in BMMCs from both WT and Mcpt5-Siglec-8 mice during the first week in culture and increased over time. Siglec-8 mRNA was also detected in BMMCs from Mcpt5-Siglec-8 mice within a week of culture, with an expression pattern that paralleled that of the Mcpt5 mRNA. As expected, no Siglec-8 mRNA was detected in WT mouse mast cells (Figure 7B). As quantitated by flow cytometry at different time points, the purity and pattern of maturation of mast cells in culture was unaffected by Siglec-8 expression (Figure 7C). Only BMMCs from Mcpt5-Siglec-8 mice expressed Siglec-8, and the surface expression kinetics correlated with that of Siglec-8 mRNA expression (Figure 7B,D,E). About 39% of mast cells expressed Siglec-8 (Figure 7E), which was consistent with <50% activation of Mcpt5-Cre in BMMC as seen in our previous work [23]. On the other hand, a much higher percentage of BMMCs expressed Siglec-8 in cells derived from CPA3-Siglec-8 mice, consistent with the broader and more consistent expression of CPA3 in BMMCs (Figure 6G). These results showed that BMMCs from Mcpt5-Siglec-8 and CPA3-Siglec-8 mice express Siglec-8 and that the expression pattern depends on the activity of the promoter used.

## 3. Discussion

Here we describe the second use of ROSA26-Siglec-8 knock-in mice containing a CAG-STOP-Siglec-8 gene and a conditional knock-in approach utilizing the Cre-LoxP system to create strains of mice in which human Siglec-8 gets expressed on subsets of mouse cells. In its first reported use, this strain was crossed with an eosinophil-specific Cre mouse, resulting in consistent cell surface Siglec-8 expression on eosinophils [19]. We now report that breeding of the ROSA26-Siglec-8 knock-in mice with either CPA3-Cre or Mcpt5-Cre transgenic mice successfully generated mouse strains in which human Siglec-8 was expressed consistently and predominantly, if not exclusively, on mast cells. After cross-breeding between ROSA26-Siglec-8 and the mast cell Cre lines, we did not notice any developmental issues in offspring that were positive for both Cre and ROSA26-Siglec-8. In multiple tissues and body compartments, Siglec-8 mRNA and/or cell surface expression was observed on most mast cells. Levels of Siglec-8 on mast cells in these mouse strains were similar to those on human skin mast cells. In vitro, Siglec-8 was expressed relatively early in BMMC development, consistent with its induction by CPA3 or Mcpt5 promoter activity. Where examined, the expression of Siglec-8 did not significantly affect baseline numbers of mast cells in vivo or the ability to generate BMMCs in vitro when compared to WT mice or littermate controls, suggesting that the expression of Cre or Siglec-8 had no adverse effect on mast cell development.

There were some potential, albeit subtle, differences between the CPA3-Cre x ROSA26-Siglec-8 mice and the Mcpt5-Cre x ROSA26-Siglec-8 mice. For example, the former mice displayed Siglec-8 expression on about 15% basophils besides mast cells, consistent with what has been reported with other mouse lines derived using the CPA3-Cre strain [21]. This is similar to the observation that Siglec-8 was seen in a small percentage of human basophils [2,4]. Although Mcpt5 is a mast cell-specific protease, Mcpt5 is not expressed in all mast cells [24], so it was not unexpected that Siglec-8 was not expressed on all mast cells in the Mcpt5-Siglec-8 mice. In a newly generated mouse strain in which Siglec-8 was targeted to eosinophils, surface Siglec-8 was functional. Engagement caused Siglec-8 to be internalized, caused tyrosine phosphorylation, and cell death [19]. It remains to be determined whether Siglec-8 on the surface of mouse mast cells leads to downstream signaling and functional consequences in mouse mast cells similar to those seen with human mast cells, such as Siglec-8-dependent inhibition of FcεRI-mediated mediator release, and inhibitory signaling that was shown to require the Siglec-8 cytoplasmic membrane-proximal immunoreceptor tyrosine-based inhibitory motif (ITIM) domain [8]. If functional, future studies in which these Siglec-8 mast cell-expressing mouse strains are crossed with strains lacking molecules downstream of putative Siglec-8 signaling pathways, such as various phosphatases, can be explored, and would provide mechanistic insights into Siglec-8 function on mast cells. Regardless of its function, molecular methods have successfully introduced Siglec-8 into the mouse mast cell compartment, and these mice will facilitate approaches to target Siglec-8 on mast cells for their elimination in vivo using strategies that we have developed and successfully tested in vitro [10]. By crossing these Siglec-8 mast cell-expressing mice with our existing Siglec-8 eosinophil-expressing mice [19], we will generate another novel strain that better matches the pattern of human Siglec-8 expression on human cells.

In summary, we have successfully established two novel mouse strains that express human Siglec-8 selectively on tissue mast cells and on BMMCs. These new mouse strains will enable in vivo studies of the behavior and function of Siglec-8 on mast cells and some basophils, particularly in disease models, including allergic responses, asthma, and other mast cell-related disorders.

## 4. Materials and Methods

### 4.1. Generation of a ROSA26-Siglec-8 Construct and Transfection of the ROSA26-Siglec-8 Construct into HEK 293T Cells

Initially two targeting DNA constructs were generated based on the pRosa26-DEST vector, a gift from Nick Hastie and Peter Hohenstein (Addgene plasmid #21189, Addgene, Cambridge, MA, USA) [25]. As shown in Figure 1, the first construct contained a ROSA26 5′ arm, the synthetic CAG promoter (a hybrid promoter construct consisting of CMV enhancer, chicken beta-actin promoter and rabbit beta-globin splice acceptor), a Neo resistant gene as a STOP fragment floxed by two LoxP sites, full length human Siglec-8 cDNA, and a ROSA26 3′ arm (CAG-Neo-ROSA26-Siglec-8). A second construct, pCMV-Neo-ROSA26-Siglec-8 (not shown), was made in the same manner except that the CMV promoter was used instead of the CAG promoter. Before generating knock-in mice, testing was performed to determine whether the Siglec-8 cDNA in the targeting construct could be properly expressed when activated by Cre recombinase (Cre) in transfected cells. HEK 293T cells (ATCC, Manassas, VA, USA) were cultured in DMEM with 10% FBS and transfected with pCAG-Neo-ROSA26-Siglec-8 or pCMV-Neo-ROSA26-Siglec-8 plasmid with or without pCAG-Cre:Green fluorescence protein (GFP) plasmid, a gift from Connie Cepko (Addgene plasmid #13776) [26], in an equal molar ratio using Lipofectamine 2000 (Invitrogen, Carlsbad, CA, USA) following the manufacturer’s instructions. Transfection and expression of Cre were confirmed by visualizing GFP fluorescence under the microscope and Siglec-8 expression was determined by western blot and flow cytometry.

### 4.2. Generation of Human Siglec-8 Conditional Knock-in Mice

All experiments and procedures performed on animals in this study were approved by the Yale University Institutional Animal Care and Use Committee (IACUC) for protocol 2016-11540 approved 21 June 2016 and by the Northwestern University IACUC for protocol IS00007627 approved 11 January 2018. All procedures were in compliance with the NIH guidelines of humane treatment of experimental animals. After confirmation of Siglec-8 expression in vitro in transfected HEK 293T cells in the presence of co-transfected pCAG-Cre:GFP (see above), the ROSA26-Siglec-8 construct with the CAG promoter (pCAG-Neo-ROSA26-Siglec-8) was used to transfect C57BL/6 mouse ES cells that were then micro-injected into blastocysts to generate the ROSA26-Siglec-8 knock-in mice. This procedure was carried out via a contract with the Transgenic Core Facility of Louisiana State University. Six chimeric mice were identified and genotyped using the Siglec-8 primers to confirm the presence of the ROSA26-Siglec-8 knock-in gene in these mice.

### 4.3. Targeting Human Siglec-8 Expression to Mouse Mast Cells

To target the expression of human Siglec-8 to mouse mast cells in vivo, ROSA26-Siglec8 (heterozygous knock-in) mice were cross-bred to two different mast cell-specific Cre transgenic mouse lines, the CPA3 (carboxypeptidase A3)-Cre mice and the Mcpt5 (mast cell protease 5)-Cre mice. The CPA3-Cre transgenic mice (a kind gift from Dr. Stephen J. Galli of Stanford University) and Mcpt5-Cre transgenic mice were generated as described previously [21,22]. From these breeding arrangements, mice with various combinations of genotypes were obtained. Comparison of Siglec-8 expression was carried out among CPA3-Cre x ROSA26-Siglec-8 mice, Mcpt5-Cre x ROSA26-Siglec-8 mice, and their control groups, i.e., ROSA26-Siglec-8, CPA3-Cre, Mcpt5-Cre, and WT mice.

### 4.4. Genotyping of ROSA26-Siglec-8 KI Mice and Cre Transgenic Mice

Genotype identification of ROSA26-Siglec-8 mice was carried out using three primers, Primer1: 5′-TTCCCCTCGTGATCTGCAAC-3′; Primer2: 5′-GCAGAGACTCAGGCCTGTTT-3′; and Primer3: 5′-TGGGAAGTCTGGTCCCTCCA-3′, using a PCR protocol with an annealing temperature of 52 °C for 30 cycles. The size of the PCR product for the WT ROSA26 allele was 167 bp and for the ROSA26-Siglec-8 knock-in allele was 394 bp. Identification of the CPA3-Cre mice was done as described previously [21] and that of the Mcpt5-Cre as described previously [22].

### 4.5. RT-PCR Analysis of Mcpt5 and Siglec-8 mRNA Expression in BMMCs

Bone marrow-derived mast cells (BMMCs) were generated from WT mice, CPA3-Cre x ROSA26-Siglec-8 mice, and Mcpt5-Cre x ROSA26-Siglec-8 mice as described below. Total mRNA was extracted from BMMCs using RNeasy Mini Kit from QIAGEN (Valencia, CA, USA) following manufacturer’s instructions. DNase I (RNase-free) digestion was performed to remove contaminating DNA in the RNA samples. To detect mRNA expression of mouse *Mcpt5* and human Siglec-8 in BMMCs, cDNA was generated using qScript cDNA synthesis kit (Quanta BioSciences, Beverly, MA, USA). RT-PCR was performed using mouse *Mcpt5* (Assay ID: Mm00487638) and human Siglec-8 (Assay ID: Hs00274289) FAM-labeled TaqMan probes (Applied Biosystems, Beverly Hills, CA, USA). The reaction was performed in an ABI 7500 thermal cycler (Applied Biosystems) for 50 cycles. Mouse *Actb* (β-actin, Assay ID: Mm02619580) was used as an internal control, and changes in the threshold cycle value for each gene of interest were displayed.

### 4.6. Antibodies and Flow Cytometric Analysis of Siglec-8 Expression in Cells and Tissues

The expression of cell surface markers was analyzed on a FACSAria II or LSR II (BD Biosciences, Waltham, MA, USA) using FlowJo data analysis software Version 8.8.6 (FlowJo, LLC, Ashland, OR, USA). Briefly, transfected cells or tissue cells were isolated, red blood cells were lysed, and single-cell suspensions were incubated with Aqua LIVE/DEAD fixable stain for 20 min and washed twice. Next, 2 × 10^5^ cells were stained with a combination of antibodies for 15 min at room temperature and analyzed by flow cytometry with gating strategies as described in the text. Fluorescent-labeled antibodies used in this study were listed in Table 1.

### 4.7. Bone Marrow-Derived Mast Cell (BMMC) Culture

Bone marrow was flushed from femurs and tibias of mice, and bone marrow cells were collected by centrifugation. Cells were cultured in BMMC media (RPMI 1640 media containing 2 mM l-glutamine, 10% FBS (Atlanta Biologicals, Flowery Branch, GA, USA), 25 mM HEPES (Sigma, St. Louis, MO, USA), 1 mM sodium pyruvate (Sigma), 0.1 mM non-essential amino acids (Sigma), 100 U/mL penicillin, 100 µg/mL streptomycin, 0.05 mM β-mercaptoethanol (Sigma), and 30 ng/mL recombinant mIL-3 (Miltenyi Biotec, Somerville, MA, USA) for 4–6 weeks. To assess the purity of BMMC after 4 weeks in culture, cells were incubated with PE–anti-mouse FcεRI (MAR-1, eBioscience, Waltham, MA, USA) and APC–anti-mouse CD117 (2B8, BD Biosciences, San Jose, CA, USA) then analyzed by flow cytometry.

### 4.8. Tissue Digestion for Mouse Mast Cell Enumeration

For skin tissue digestion, ear skin and back skin were harvested and separated into ventral and dorsal halves. Tissue was then placed in HBSS with 2 U/mL Liberase^™^ (Roche, Indianapolis, IN, USA) and minced with scissors. Homogenates were incubated for 30 min at 37 °C with constant agitation. Digestion was stopped with the addition of cold 10% fetal bovine serum and samples were put through a 70 μm strainer. Total cell numbers were counted and then labeled for flow cytometry. Cells from lung, tongue, trachea, and esophagus were isolated according to ImmGen protocols (http://www.immgen.org/Protocols/ImmGen%20Cell%20prep%20and%20sorting%20SOP.pdf) and as previously described [27]. Briefly, tissues were minced finely between two scalpel blades and placed in digestion buffer (RPMI with 10% FBS, 600 U/mL collagenase IV (Worthington), 0.1% dispase (Gibco, Grand Island, NY, USA) and 20 μg/mL DNase I (Roche)). Minced tissues were incubated for 30 min at 37 °C with constant agitation at 300 RPM. Single cell suspensions from spleens were obtained by compressing the spleens between the frosted sections of glass slides. Red blood cells were lysed, and the samples were filtered prior to labeling for flow cytometry.

### 4.9. Human Skin Mast Cell Isolation and Culture

Human skin mast cells were isolated as previously described [28]. In brief, discarded surgical skin samples were obtained from the Skin Disease Research Center (Northwestern University) or purchased from the Cooperative Human Tissue Network (CHTN) for processing as approved by the Institutional Review Board (IRB) at Northwestern University. The subcutaneous fat layer was removed, and samples were minced and digested in wash buffer (Hanks balanced salt solution, 0.035% sodium bicarbonate, 1% FCS, 10 mM HEPES, 0.05% amphotericin B and 1% penicillin and streptomycin) supplemented with DNase type 1 (0.15 mg/mL), hyaluronidase (0.7 mg/mL), type 2 collagenase (592.5 U/mL) and 1 mM CaCl_2_. Samples were digested at 37 °C for 1 h. The mixture was filtered through a wire mesh and the tissue was collected for additional digestion, while the collected flow-through was washed, filtered through a 40-micron filter and washed again. After 3 digestions, the collected pellets were combined and processed through a Percoll gradient. Cells were collected, washed and plated in 24-well plates at a concentration of 1 × 10^6^ cells/well/2 mL of serum-free X-Vivo or Aim V media supplemented with 100 ng/mL recombinant human SCF. Cells were fed weekly and maintained at a concentration of 5 × 10^5^ cells/mL, splitting as necessary.

### 4.10. Western Blot

After plasmid construct transfection and incubation, proteins were extracted from HEK 293T cells by RIPA buffer, separated by 4–12% Bis-Tris gel (Invitrogen) and transferred onto a PVDF membrane (Invitrogen) by iBlot (Thermo Fisher Scientific, Waltham, MA, USA). After blocking with 5% nonfat dry milk for 1 h at room temperature, the membranes were incubated at 4 °C overnight with sheep anti-Siglec-8 antibody as primary antibody (a generous gift from Dr. Paul Crocker, University of Dundee), and then 1 h with anti-sheep IgG conjugated with alkaline phosphatase (AP) (ab6901, Abcam, Cambridge, MA, USA), as secondary antibody. Vector Red Substrate (Vector Laboratories, Burlingame, CA, USA) was used as substrate to visualize the target bands. Photographs of the gels were taken using the PXi imaging system by Syngene (Frederick, MD, USA).

### 4.11. Statistical Analysis

Data were analyzed using GraphPad Prism 6 software (GraphPad Software, La Jolla, CA, USA) using two-tailed Student’s *t* test or two-way ANOVA to determine significance. Differences between groups of samples were considered statistically significant when *p* <0.05. (* *p* < 0.05, ** *p* < 0.01, *** *p* < 0.001, and **** *p* < 0.0001).

## Figures and Tables

**Table 1:** Antibody Panel Information.

Antibody	Clone	Fluorophore Conjugation	Source
Anti-Siglec-8 (mouse IgG1)	2E2	Alexa Fluor 488	Produced in house
Mouse IgG1 Isotype	MOPC-21	Unconjugated, Alexa Fluor 647 or Alexa Fluor 488	Tonbo Biosciences, San Diego, CA, USA Conjugated to Alexa Fluor 488 in house
Anti-CD3	500A2	Pacific Blue	Invitrogen
Anti-CD11b	M1/70	APC-Cy7	BD Pharmingen
Anti-CD11c	N418	APC	Biolegend
Anti-CD19	1D3	PE	BD Biosciences
Anti-CD45	1O4	PE-Cy7	BD Pharmingen
Anti-CD45	30-F11	APC-Cy7	BD Pharmingen
Anti-CD49b	DX5	PE	BD Pharmingen
Anti-CD117 (c-Kit)	2B8	BV421	BD Pharmingen
Anti-CD117 (c-Kit)	2B8	APC	eBioscience
Anti-CD117 (c-Kit)	2B8	FITC	eBioscience
Anti-FcεRI	MAR-1	PE-Cy7	Biolegend
Anti-FcεRI	MAR-1	PE-Cy7	eBioscience
Anti-FcεRI	MAR-1	APC	Biolegend
Anti-Ly-6G and Ly-6C (Gr-1)	RB6-8C5	Pacific Blue	Invitrogen
Anti-Ly-6G and Ly-6C (Gr-1)	RB6-8C5	eFluor450	eBioscience
Anti-Siglec-F	E50-2440	PE	BD Pharmingen
Anti-Siglec-8 (mouse IgG1)	2C4	Alexa Fluor 647	Produced in house
Anti-Siglec-8 (mouse IgG1)	7C9	PE	Biolegend
Anti-Siglec-8 (mouse IgG1)	7C9	APC	Biolegend

## Abbreviations

BMMC	Bone marrow derived mast cell
CAG promoter	A hybrid promoter consisting of CMV enhancer, chicken β-actin promoter and rabbit β-globin splice acceptor
CPA3	Carboxypeptidase A3
Cre	Cre recombinase
ES cells	Embryonic stem cells
GFP	Green fluorescence protein
KI	Knock-in
Mcpt5	Mast cell protease 5
pCAG-Neo-ROSA26-Siglec-8	ROSA26-Siglec-8 construct with the CAG promoter
pCMV-Neo-ROSA26-Siglec-8	ROSA26-Siglec-8 construct with the CMV promoter
